# Comparison of Thyroid Hormone Levels Between Patients With Major Depressive Disorder and Healthy Individuals in China

**DOI:** 10.3389/fpsyt.2021.750749

**Published:** 2021-10-14

**Authors:** Yanan Zhou, Yuejiao Ma, Qiuxia Wu, Qianjin Wang, Winson Fu Zun Yang, Yunfei Wang, Dong Yang, Yinli Luo, Kewei Tang, Tieqiao Liu, Dongfang Wang

**Affiliations:** ^1^Department of Psychiatry, Hunan Brain Hospital (Hunan Second People's Hospital), Changsha, China; ^2^Department of Psychiatry, Second Xiangya Hospital, Central South University, Changsha, China; ^3^Department of Psychological Sciences, College of Arts and Sciences, Texas Tech University, Lubbock, TX, United States; ^4^Changqiao Compulsory Isolation and Rehabilitation Center, Changsha, China; ^5^Department of Psychiatry and Psychotherapy, University Hospital Rechts der Isar, Technical University of Munich, Munich, Germany

**Keywords:** thyroid hormones, major depressive disorder, FT3, FT4, TSH

## Abstract

Abnormal thyroid function in major depressive disorder (MDD) has been studied extensively, but the results still remain inconsistent. In China, few large-scale studies have investigated the differences in the levels of thyroid hormones between patients with MDD and healthy controls (HCs). In this retrospective, cross-sectional study, 535 MDD patients and 998 HCs were included. We compared the levels of thyroid hormones (FT3, FT4, and TSH) between the two groups, as well as investigated the distribution of levels of thyroid hormones within and outside normal ranges. The results showed that all the three hormones were significantly lower in MDD patients than in HCs, which was also true in different gender and age subgroups. The proportion of subjects with levels of all the three hormones outside the normal range in the MDD group was higher than that in the HC group (all *p* < 0.05). However, no significant difference was found in clinical/subclinical hyperthyroidism or hypothyroidism between the two groups (*p* > 0.05). Our study showed that the levels of thyroid hormones were lower in MDD patients, suggesting that there was an association between abnormal thyroid function and depression. The higher rate of thyroid dysfunction in MDD patients indicated the importance of regular monitoring of thyroid function.

## Introduction

Major depressive disorder (MDD) is one of the most common mood disorders across the world. As a chronic, recurrent, and disabling disorder, it leads to a decline in the quality of life of patients (1, 2) and accounts for a large part of the global burden of mental illness (3–5). The global point prevalence of MDD was 4.7% (4.4–5.0%) (6), while in China, it was about 6.0% (7).

There are multiple causes for depression, which have not been fully understood. The abnormality of the neuroendocrine system, especially the hypothalamic-pituitary-thyroid (HPT) axis, has been reported in studies on depressive disorders and attracted extensive attention (8, 9). For the assessment of the HPT axis function, thyroid hormones (including basal TSH, FT3 and FT4 plasma levels) were considered to be more accurate and suitable indicators, as compared with the thyrotropin releasing hormone (TRH) (10). Thus, many studies focused on the peripheral blood thyroid hormones when studying depressive disorder.

The association between thyroid function and depression has long been recognized. On one hand, both insufficient and excess thyroid hormones can be accompanied by a variety of neuropsychiatric manifestations including depression. Some studies have reported that patients with overt hypothyroidism exhibited some depressive symptoms (11–13). Chueire et al. found that the prevalence of MDD was more than four times (OR = 4.9) in subclinical hypothyroidism patients than in the general population (14). Zhou et al. found that the function of thyroid hormones was associated with suicide attempts in MDD patients with anxiety symptoms (15). On the other hand, depression may be accompanied by mild thyroid dysfunction. It has been reported that subclinical hypothyroidism is very common in people with refractory depression and simple depression (16, 17), although overt thyroid diseases in patients with depression were less reported (18). The evidence of thyroid dysfunction in depressed patients includes changes in serum T3 or T4 levels (although mostly in the normative range), blunted TSH response to TRH, or excessive TSH response to TRH (19, 20).

Despite some studies indicating a link between thyroid function and depression, other studies failed to reveal this association. For example, using a large sample of 30,589 subjects, Engum et al. found no statistical relationship between various degrees of thyroid dysfunction and the presence of depression (21). A recent study using data of 3,932 elderly males without overt thyroid dysfunction also indicated no connection between subclinical thyroid disease and incident depression (22). Hence, the interaction between thyroid function and depression still remains unclear and needs to be further explored.

In the present study, we used peripheral blood thyroid hormones to investigate the association between thyroid function and MDD in a population-based cross-sectional study. Using data from a relatively large sample, we aim to: (1) compare the level of thyroid hormones (TSH, FT3, and FT4) between MDD patients and healthy controls (HCs); (2) investigate the distribution of thyroid hormones within and outside normal ranges; and (3) find out the difference in thyroid hormone levels in different gender and age cohorts.

## Methods

### Study Procedure

This retrospective, cross-sectional study included data from 1,535 subjects aged 16–65 years. After screening, data of 537 consecutive MDD patients from the database of the Second Xiangya Hospital of Central South University between August 1, 2015 and April 30, 2017 were selected as the MDD group. MDD was diagnosed by at least two trained psychiatrists using *the Diagnostic and Statistical Manual of Mental Disorders, Fifth Edition* (*DSM-V*). Patients with diagnosed thyroid diseases, pregnancy, or other psychiatric disorders identified by medical records were excluded. Nine hundred ninety-eight HCs were also recruited among community residents and employees of this hospital who underwent periodic health examinations at the Second Xiangya Hospital Healthcare System from January 1, 2017 to May 20, 2017. The HCs met the following criteria: (1) aged 16–65 years; (2) with no diagnosed psychiatric disorders or thyroid diseases identified by medical records; (3) with test for thyroid hormones included in their health examination. For each subject, two members of the study team manually collected information about gender, age, levels of thyroid hormones (including TSH, FT3, and FT4), etc. This study was approved by the Ethics Committee of the Second Xiangya Hospital of Central South University.

### Thyroid Function

Blood samples were collected at 6–7 a.m. on the next day after admission for the MDD patients, and in the morning of the physical examination for HCs. All the samples were tested immediately with Chemiluminescent Microparticle Immunoassay (CMIA) for the quantification of serum and plasma TSH, FT3, and FT4. The thyroid hormones were analyzed at the nuclear medicine laboratory of the Second Xiangya Hospital using Architect i2000 SR analyzer (Abbott, Longford, Ireland). The reference ranges at the local laboratory are as follows: TSH: 0.35–4.94 μIU/ml, FT3: 2.63–5.70 pmol/l, and FT4: 9.01–19.05 pmol/l. The Architect TSH assay is designed to have a precision of ≤ 10% (total analytical variation), functional sensitivity of ≤ 0.0036 μIU/ml (95% CI: 0.0034–0.0038), and analytical sensitivity of ≤ 0.0025 μIU/ml. The FT3 assay has a total analytical variation of ≤ 10% and analytical sensitivity of ≤ 1.536 pmol/l. The total analytical variation of the assay for FT4 was ≤ 10%, and the FT4 assay is designed to have a limit of quantitation (LQQ) of ≤ 5.148 pmol/l.

### Statistical Analysis

Statistical analyses were performed using IBM SPSS statistics (version 20.0), and graphs were constructed using Graph Pad Prism version 7.0. Simple descriptive statistics (median and quartiles) were generated for continuous variables, except for age (means and standard deviations). As the three variables (FT3, FT4, and TSH) were not normally distributed even after transformation, Kruskal-Wallis test was used for inter-group comparison, and Wilcoxon rank-sum test was used for intra-group comparison. Proportions were compared using chi-square test. Correlation analyses were performed using Spearman's rank correlation coefficients. The threshold of statistical significance for all tests was set at *p* < 0.05.

## Result

### Demographic Features

A total of 1,535 subjects were included in this study. The proportion of females was 62.60% (*n* = 336) in the MDD group (*n* = 537) and was 55.50% (*n* = 554) in the HC group (*n* = 998); the proportion of males was 37.40% (*n* = 201) in the MDD group, and was 44.50% (*n* = 444) in the HC group. A significant gender difference was observed between the two groups (χ^2^ = 7.14, *df* = 1, *p* = 0.008). There was no difference in age (M ± SD) between the MDD group (36.15 ± 13.59 years) and the HCs (36.60 ± 12.16 years) (*Z* = −1.17, *p* = 0.244).

The samples for serum TSH level were collected from 536 MDD patients (99.81%, 201 males, 335 females, with missing data for one subject) and 998 HCs (with no missing data). The samples for serum FT3 and FT4 levels were collected from 525 MDD patients (97.77%, 195 males, 330 females, with missing data for 12 subjects) and 998 HCs (with no missing data).

Statistical analysis revealed a significant difference in the proportion of subjects with normal levels of thyroid hormones (TSH, FT3, FT4) between the two groups (χ^2^ = 14.41, *df* = 1, *p* < 0.0001), with 75.38% (*n* = 395) of the MDD patients and 83.47% (*n* = 833) of the HCs having normal levels of thyroid hormones. The proportion of subjects with decreased TSH levels and elevated FT3 and FT4 levels was 0.76% in the MDD group and 0.10% in the HC group, with no significant difference between the two groups (χ^2^ = 4.62, *df* = 1, *p* = 0.051). The overall rate of subclinical hyperthyroidism, which is defined as low TSH levels with normal FT3 and FT4 levels, was 1.34% in the MDD group and 0.40% in the HC group, with no significant difference between the two groups (χ^2^ = 4.19, *df* = 1, *p* = 0.055). There was also no significant difference between the two groups regarding the rate of subclinical hypothyroidism which is characterized by the presence of elevated TSH levels with normal FT3 and FT4 levels (χ^2^ = 0.15, *df* = 1, *p* = 0.283). We did not find any subject with high TSH with low FT3 and FT4 levels in the two groups.

### Results of TSH Assay

We found that the serum TSH level in the MDD group was significantly lower than that in the HC group (*Z* = −10.68, *p* < 0.0001), and the same result was found for the subgroups of males (*Z* = −6.56, *p* < 0.0001) and females (*Z* = −9.14, *p* < 0.0001). We further found that the serum TSH level in males was significantly lower than that in females in the MDD group (*Z* = −2.64, *p* = 0.008) as well as in the HC group (*Z* = −7.53, *p* < 0.0001) (Table 1).

**Table 1:** Levels of thyroid hormones by gender in the MDD group and the HC group.

	**TSH**			**FT3**			**FT4**		
	**Male**	**Female**		**Male**	**Female**		**Male**	**Female**	
MDD	1.45 (1.07, 2.13)	1.63 (1.13, 2.47)	*Z* = −2.64 *P* = 0.008	4.95 (4.56, 5.44)	4.36 (3.82, 4.88)	*Z* = −8.56 *p* < 0.0001	14.96 (13.45, 16.81)	14.81 (12.87, 16.79)	*z* = −0.91 *P* = 0.361
HCs	2.00 (1.45, 2.61)	2.44 (1.73, 3.67)	*Z* = −7.53 *p* < 0.0001	4.93 (4.62, 5.24)	4.55 (4.21, 4.93)	*Z* = −10.96 *p* < 0.0001	16.0.86 (15.57, 18.24)	16.08 (14.80, 17.37)	*Z* = −6.19 *p* < 0.0001
	Z = −6.56 *p* < 0.0001	Z = −9.14 *p* < 0.0001	–	*Z* = −0.95 *P* = 0.342	Z = −4.04 *p* < 0.0001	-	*Z* = −8.37 *p* < 0.0001	Z = −6.09 *p* < 0.0001	-

*MDD, major depressive disorder, HCs, healthy controls*.

**Figure 1:**
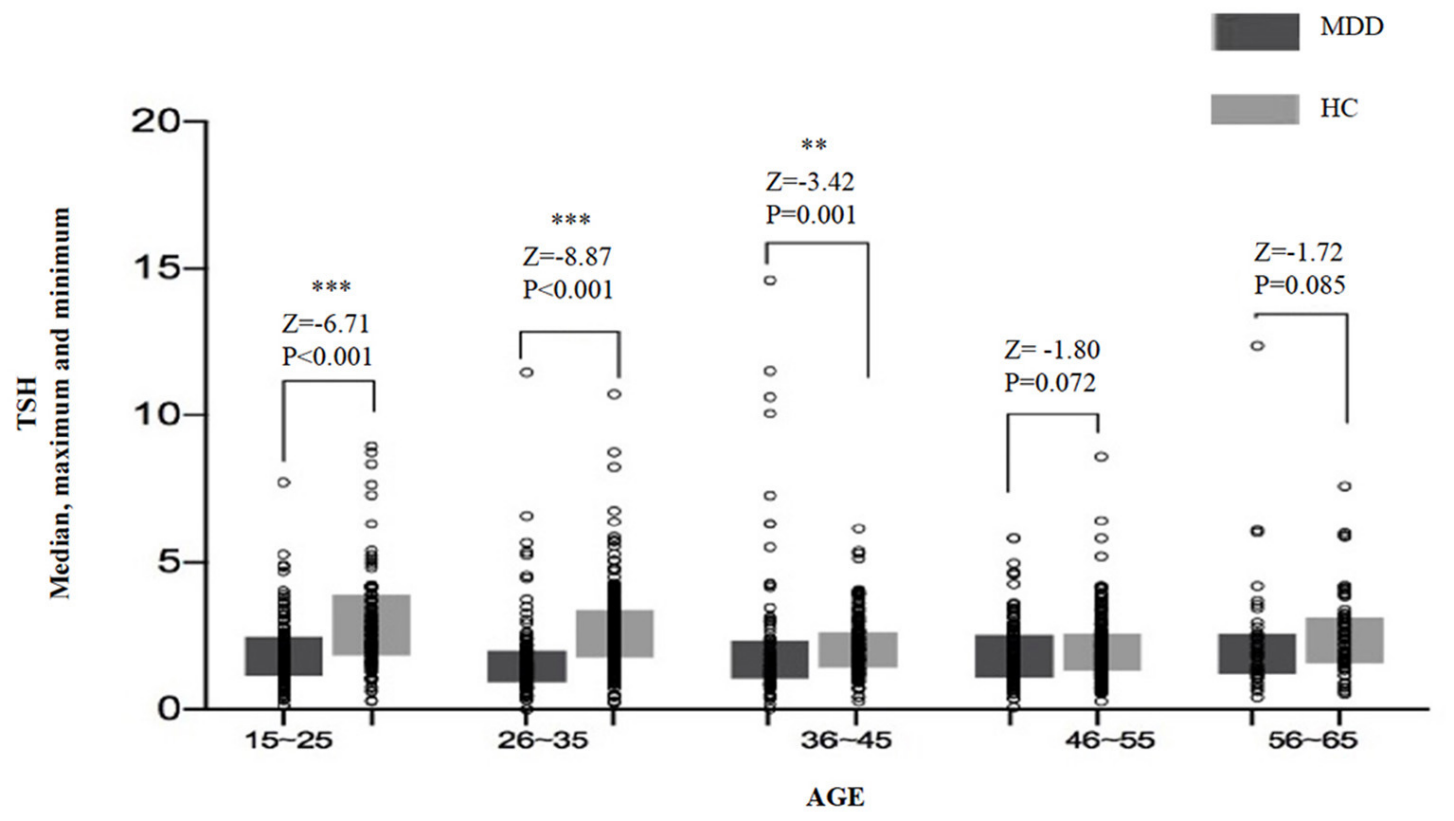
The median, maximum, and minimum values of the TSH level (*IU/ml*) at different ages in the MDD and HC groups. * <0.05, ** <0.01.

For the analysis of TSH levels, all the subjects were divided into five age subgroups: 16–25, 26–35, 36–45, 46–55, and 56–65 years. The TSH levels of different age subgroups in the two groups are shown in Figure 1. Significant differences in TSH levels were found between age subgroups in the MDD group (*H* = 10.20, *df* = 4, *p* = 0.037) as well as in the HC group (*H* = 58.44, *df* = 4, *p* < 0.0001). Compared with the HC group, the TSH level was significantly lower in three subgroups (i.e., 16–25, 26–35, and 36–45 years) of MDD patients, except for the subgroups aged 46–55 years (*Z* = −1.80, *p* = 0.072) and 56–65 years (*Z* = −1.72, *p* = 0.085) (Table 2).

**Table 2:** Median and quartile of thyroid hormones levels in age subgroups.

**Thyroid**		**Age subgroups**						
		**Total**	**16–25**	**26–35**	**29–45**	**46–55**	**56–65**	**K-W**
TSH	MDD	1.52 (1.09, 2.36)	1.69 (1.14, 2.46)	1.38 (0.93, 1.99)	1.47 (1.05, 2.33)	1.53 (1.08, 2.52)	1.89 (1.19, 2.53)	*H* = 10.20 *P* = 0.037
	HCs	2.20 (1.58, 3.14)	2.52 (1.83, 3.90)	2.33 (1.76, 3.40)	2.05 (1.41, 2.64)	1.88 (1.31, 2.58)	2.2 (1.56, 3.15)	*H* = 58.44 *p* < 0.0001
	M-U	*Z* = −10.68	*Z* = −6.71	*Z* = −8.78	*Z* = −3.42	*Z* = −1.80	*Z* = −1.72	-
		*p* < 0.0001	*p* < 0.0001	*p* < 0.0001	*p* < 0.0001	*P* = 0.072	*P* = 0.085	
FT3	MDD	4.65 (4.09, 5.13)	4.79 (4.28, 5.36)	4.77 (4.09, 5.25)	4.47 (3.98, 5.06)	4.45 (3.92, 5.00)	4.45 (4.11, 4.81)	*H* = 14.79 *P* = 0.005
	HCs	4.75 (4.38, 5.10)	4.74 (4.39, 5.13)	4.72 (4.33, 5.11)	4.85 (4.47, 5.16)	4.77 (4.45, 5.04)	4.50 (4.28, 4.76)	*H* = 15.87 *P* = 0.003
	M-U	Z = −3.47	Z = −0.67	Z = −0.36	Z = −3.53	Z = −3.63	Z = −0.64	-
		*p* < 0.0001	*p =* 0.950	*p =* 0.72	*p* < 0.0001	*p* < 0.0001	*p =* 0.525	
FT4	MDD	14.9 (13.26, 16.80)	14.44 (13.74, 16.04)	14.83 (13.13, 16.86)	15.05 (13.51, 16.86)	15.2 (13.22, 17.11)	16.15 (13.78, 18.83)	*H* = 13.85 *P* = 0.008
	HCs	16.34 (15.15, 17.89)	16.47 (15.32, 18.02)	16.34 (15.06, 17.63)	16.60 (15.19; 18.21)	16.22 (15.06, 17.23)	16.15 (14.51, 17.63)	*H* = 7.28 *P* = 0.122
	M-U	*Z* = −10.06	*Z* = −7.82	*Z* = −4.67	*Z* = −4.28	*Z* = −3.80	*Z* = −0.23	-
		*p* < 0.0001	*p* < 0.0001	*p* < 0.0001	*p* < 0.0001	*p* < 0.0001	*p =* 0.820	

*MDD, major depressive disorder, HCs, healthy controls*.

*Intra-group comparison of age subgroups, Kruskal–Wallis test*.

*Inter-group comparison of the MDD and HC groups, Wilcoxon rank-sum test*.

The proportion of subjects with normal TSH levels (0.35–4.94 mIU/L) was not significantly different between two groups (χ^2^ = 2.67, *df* = 1, *p* = 0.102). The proportion of subjects with TSH levels outside the reference range was 5.97% in the MDD group and 4.2% in the HC group, with no significant difference between the two groups. However, the proportion of subjects with TSH levels below the reference range was 2.05% for the MDD group and 0.7% for the HC group, with a significant difference between the two groups (χ^2^ = 5,49, *df* = 1, *p* = 0.019). No significant difference was found in the proportion of subjects with TSH levels above the normal range between the two groups (χ^2^ = 0.26, *df* = 1, *p* = 0.608) (Table 3).

**Table 3:** The distribution of levels of thyroid hormones within and outside the normal range in the MDD and HC groups.

	**TSH**			**FT3**			**FT4**		
	**<0.35**	**0.35–4.94**	**>4.94**	**<2.63**	**2.63–5.7**	**>5.70**	**<9.01**	**9.01–19.05**	**>19.05**
MDD	11 (2.05%)	504 (94.03%)	21 (3.92%)	9 (525; 1.70%)	467 (89.00%)	49 (9.30%)	6 (525; 1.14%)	460 (87.62%)	59 (11.24%)
HCs	7 (0.70%)	957 (95.89%)	34 (3.50%)	3 (998; 0.30%)	946 (94.80%)	49 (4.90%)	0 (998; 0.00%)	898 (89.98%)	100 (10.02%)
	χ^2^ = 5.49	χ^2^ = 2.67	χ^2^ = 0.26	χ^2^ = 8.80	χ^2^ = 17.49	χ^2^ = 11.18	χ^2^ = 11.45	χ^2^ = 1.99	χ^2^ = 0.25
	*p =* 0.019	*p =* 0.102	*p =* 0.608	*p =* 0.003	*p =* 0.0001	*p =* 0.001	*p =* 0.002	*p =* 0.159	*p =* 0.460

*MDD, major depressive disorder, HCs, healthy controls*.

### Results of FT3 Assay

Compared to the HC group, the MDD group had a significantly lower serum FT3 level (*Z* = −3.47, *p* < 0.0001), and the difference was also significant for the subgroups of females (*Z* = −4.04, *p* < 0.0001), but not for males (*Z* = −0.95, *p* = 0.342). As shown in Table 1, the FT3 level of males was significantly higher than that of females in both the MDD group (*Z* = −8.56, *p* < 0.0001) and the HC group (*Z* = −10.96, *p* < 0.0001) (Table 1).

**Figure 2:**
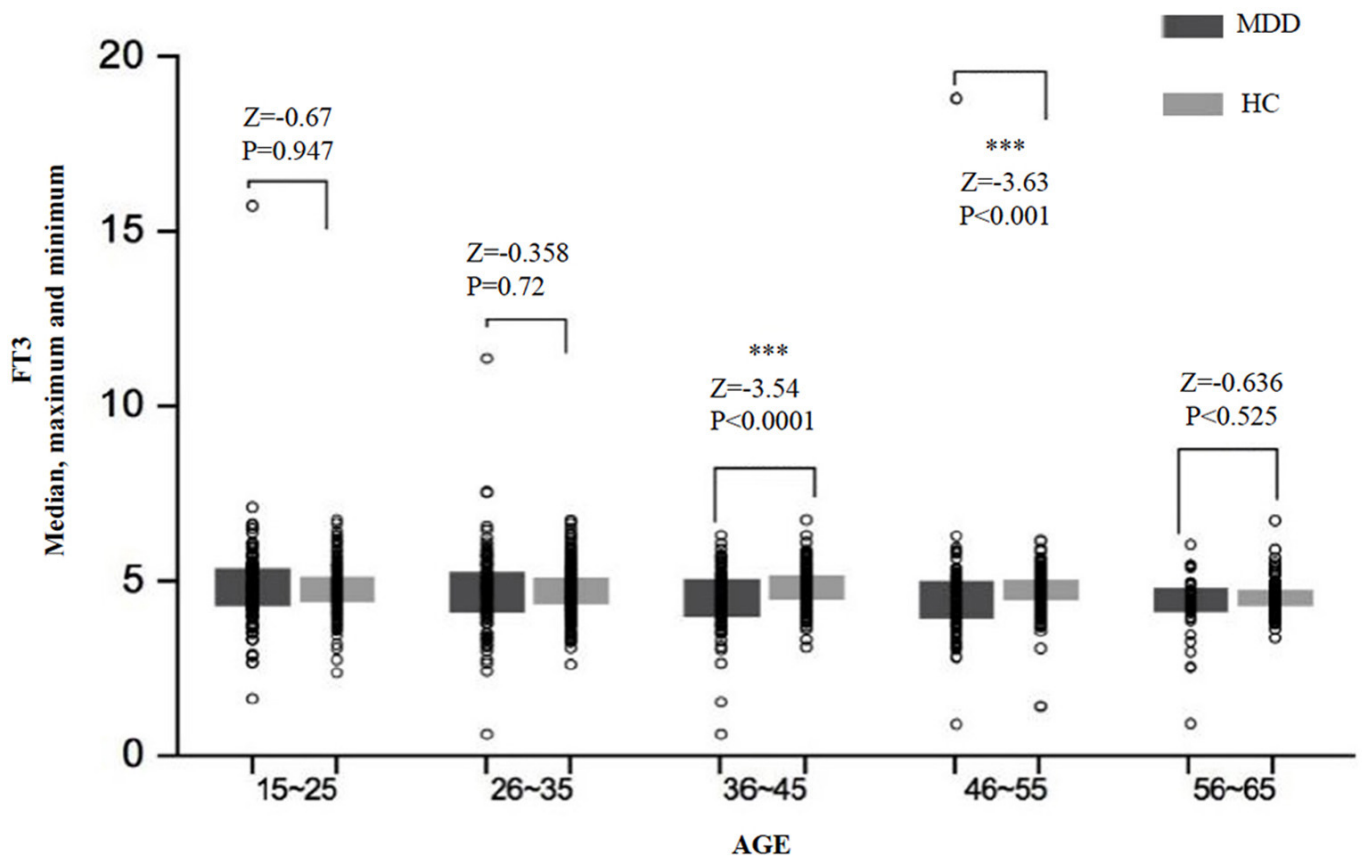
The median, maximum, and minimum values of the FT3 level (*pmol/L*) at different ages in the MDD and HC groups. *** < 0.001.

There was a significant difference in the FT3 level between age subgroups both in the MDD group (*H* = 14.79, *df* = 4, *p* = 0.005) and the HC group (*H* = 15.87, *df* = 4, *p* = 0.003) (Table 2). Comparing the MDD and HC groups, the difference in the FT3 level was significant for the subgroups of 29–45 years (*Z* = −3.53, *p* < 0.0001) and 46–55 years (*Z* = −3.63, *p* < 0.0001), but was not significant for the subgroups of 16–25 years (*Z* = −0.67, *p* = 0.950), 26–35 years (*Z* = −0.36, *p* = 0.720), and 56–65 years (*Z* = −0.64, *p* = 0.525) (Figure 2).

The proportion of subjects with normal FT3 levels (2.63–5.7 pmol/L) was significantly different between the two groups (χ^2^ = 17.49, *df* = 1, *p* < 0.0001). The proportions of subjects with FT3 levels above (>5.7 pmol/L) or below (<2.63 pmol/L) the normal range were higher in patients with MDD (11.00%) than in HCs (5.20%). The proportion of subjects with the level below the normal range was 1.70% in the MDD group and 0.30% in the HC group, with a significant difference between the two groups (χ^2^ = 8.80, *df* = 1, *p* = 0.003); the proportion of subjects with the level above the normal range was 9.30 and 4.90% in the MDD group and the HC group, respectively, with a significant difference between the two groups (χ^2^ = 11.18, *df* = 1, *p* = 0.001) as well (Table 3).

### Results of FT4 Assay

The serum FT4 level in the MDD group was significantly lower than that in the HC group (*Z* = −10.06, *p* < 0.0001), and the difference was significant for the subgroups of males (*Z* = −8.37, *p* < 0.0001), but not for females (*Z* = −6.09, *p* < 0.0001). The difference between males and females in the HC group was statistically significant (*Z* = −6.19, *p* < 0.0001), with a higher FT4 level in females compared to males. But no gender difference was found in the MDD group (*z* = −0.91, *p* = 0.361) (Table 1).

**Figure 3:**
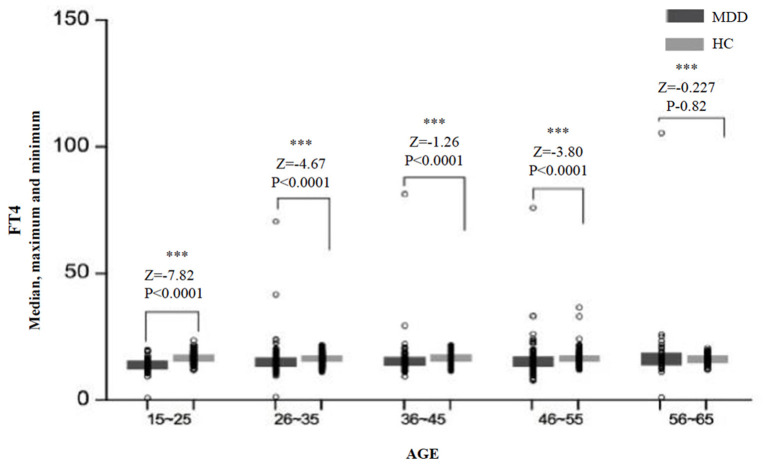
The median, maximum, and minimum values of the FT4 level (*pmol/L*) at different ages in the MDD and HC groups. *** < 0.001.

Significant differences in the FT4 level were found between age subgroups in the MDD group (*H* = 13.85, *df* = 4, *p* = 0.008) and the HC group (*H* = 7.28, *df* = 4, *p* = 0.122). An overview of serum FT4 levels in different age subgroups is presented in Figure 3. The levels of FT4 in the MDD group were significantly lower than that in the HC group for almost all the age subgroups, except for the subgroup of 56–65 years (*Z* = −0.23, *p* = 0.820) (Table 2).

There was no significant difference in the proportion of subjects with normal FT4 levels (9.01–19.05 pmol/L) between the two groups (χ^2^ = 1.99, *df* = 1, *p* = 0.159). The proportion of subjects with abnormal FT4 levels (above or below the normal range) was 12.38% in the MDD group and 10.02% in the HC group. The proportion of subjects with FT4 levels below the normal range (<9.01 pmol/L) in the MDD group (1.14%) was significantly higher than that in the HC group (0%) (χ^2^ = 11.45, *df* = 1, *p* = 0.002). However, no significant difference was found between the two groups regarding the proportion of subjects with FT4 levels above the normal range (χ^2^ = 0.25, *df* = 1, *p* = 0.46) (Table 3).

## Discussion

The majority of MDD patients in this study had normal TSH, FT3, and FT4 levels. However, there were more MDD patients with at least one of the three indicators outside the normal range (25.62%), as compared to HCs (12.53%), which was consistent with previous report in Chinese depressed population (21.97%) (23). Interestingly, inconsistent with some previous studies, we did not find any significant difference in clinical/subclinical hyperthyroidism or hypothyroidism between the two groups (24, 25). Demartini et al. found that the level of depression was significantly higher in patients with subclinical hypothyroidism than in HCs (24). In a population-based study, Kvetny et al. found that individuals with subclinical hyperthyroidism seemed to have a higher risk for depression (25). Sintzel et al. pointed out that the incidence of subclinical hypothyroidism in patients with simple depression was 8–17%, and up to 52% among patients with refractory depressions (17). The above findings supported the association between subclinical thyroid diseases and depression, which was opposite to our result. However, in line with our findings, Almeida et al. supported the relationship between subclinical thyroid diseases and clinically depressive symptoms (22). There are several possible reasons for these differences. First, we investigated three indicators, and defined thyroid dysfunction as decreased or elevated FT3 or FT4 levels with abnormal TSH levels rather than abnormal TSH levels alone. Second, as opposed to studies on the level of depression based on a population of subclinical hypothyroidism or normal population, our study underlined abnormal thyroid function in depressed patients. Third, different reference ranges for thyroid hormones in different studies have made it difficult to make a direct comparison between studies.

We also found that serum TSH, FT3, and FT4 levels in MDD patients were significantly lower than those in HCs, which was also true in male and female subgroups. We also analyzed differences in thyroid hormones between different age subgroups and found that in most subgroups, the levels of thyroid hormones in MDD patients were significantly lower than those in HCs, although results for some subgroups were not statistically significant.

Our findings of lower TSH levels in MDD patients corroborated previous findings (26, 27). Wysokiński et al. found a lower basal TSH in MDD patients, as opposed to those with other mental disorders. Several studies have shown that compared to HCs, TSH response to TRH were blunted in patients with depression (28, 29). Previous studies also found lower FT3 levels in depressed patients (18, 30). However, Kirkegaard et al. did not find any alteration in FT3 levels in depressed patients (31). Premachandr et al. noted that the total serum T3 tended to fall in patients with depression and indicated that the reduced serum T3 might be attributed to reduced TSH levels. A possible reason for this result is that the serum FT3 level can be affected by many factors, such as hunger, malnutrition, concurrent clinical diseases, and the use of antidepressants (32).

One unanticipated finding was that the FT4 level was lower in the MDD group than in the HC group. This is contradictory to most previous studies, where T4 and FT4 levels were reported to be normal (33) or higher (26, 34) in depressed patients (19, 20, 35). However, our findings are consistent with some literature reports (36, 37). In addition, it is worth mentioning that a recent study showed that low FT4 levels were associated with suicidal behavior in patients with depression (38). Another interesting study testing hair thyroid hormone concentration in Chinese females found that the T4 level increased in those in a pre-disease depressive episode and then decreased in depressive episode, as compared to healthy subjects (39). A possible reason for the inconsistencies is regional differences, as the studies supporting our conclusion is predominantly those using data from a Chinese population. Another possible explanation might be the use of FT4, instead of TT4, in our study (19, 20).

We also found gender-related differences in thyroid hormones. In our study, the level of TSH was significantly higher among females than males, while the levels of FT3 and FT4 were lower among females than males. This is in line with the finding of a previous research that the TSH level in female patients was higher than that in male patients with depression, even though the difference was not significant (27). A large population-based study also reported that the mean serum TSH level was higher in females than males (40). A possible explanation for the lower TSH level in males is that males are more likely to have blunted response to TRH stimulation (41). In addition, the negative correlation between serum FT3 and FT4 levels and the TSH level might explain why FT3 and FT4 levels were lower among females.

In the present study, we found that the MDD group had a higher proportion of subjects with hormone levels outside the normal range than the HC group. However, statistical significance was only found in the proportion of subjects with abnormal serum FT3 levels (reference range: 2.63–5.7 pmol/l) between the two groups. This was contradictory to a previous study, which found that the proportion of subjects with abnormal TSH levels (reference range: 0.4–5.0 μIU/ML) was 8.30%, higher than 5.97% in our study (42). This slight inconsistency may be explained by the different reference ranges used in the two studies. Our study also found that patients with MDD are significantly more likely to have hormone levels below the normal range regarding the three indicators, and MDD patients had significantly lower levels of the three thyroid hormones than healthy individuals.

This study had some limitations. First, we adopted a retrospective and natural experimental design for this study; thus, our data only represented the present condition and could not explain the causal relationship between thyroid dysfunction and depression. Second, according to the medical records, subjects in the HC group did not have diagnosed thyroid diseases and mental disorders; however, the diseases could not be completely ruled out at the time of sampling. Therefore, the HC group in our study could only represent a relatively healthy population. Third, for the MDD group, no detailed information regarding their medication (anti-depressants may affect thyroid function) (43) was obtained, and data of other laboratory tests for thyroid function, such as tests for T3, T4, and anti-thyroid autoantibodies, were not available. Additionally, the percentage of females in the MDD group was significantly higher than that in the HC group, which might affect the outcome to some extent. Despite these limitations, our study maintained some strengths. We included a large sample with clear MDD diagnoses in the study and used multiple indicators (serum TSH, FT3, and FT4 levels) to determine thyroid dysfunction. In this study, we not only conducted inter-group comparison between the MDD patients and HCs but also conducted inter-group and intra-group comparisons based on gender and age subgroups. All the strengths can contribute to a better understanding of the relationship between depression and thyroid function.

## Conclusion

In summary, our study showed that MDD patients exhibited higher incidence of abnormal thyroid function, as compared to HCs. However, the thyroid dysfunction here may not be defined as clinical/subclinical hypothyroidism or hyperthyroidism. We also found that the serum levels of three thyroid hormones (TSH, FT3, and FT4) were lower in MDD patients than in healthy subjects, but this needs further study on the specific mechanism. According to the results, we believe that regular monitoring of thyroid function is important for the prevention of thyroid disease as well as depression.

## Data Availability Statement

The raw data supporting the conclusions of this article will be made available by the authors, without undue reservation.

## Ethics Statement

The studies involving human participants were reviewed and approved by the Second Xiangya Hospital of Central South University. The patients/participants provided their written informed consent to participate in this study.

## Author Contributions

TL, YM, and YZ conceptualized and designed the study. YM and YZ collected the data. YZ and DW performed the statistical analyses and prepared the first draft of this manuscript. YL, DY, and KT assisted in the interpretation of results. QiuW, QiaW, YW, and WY revised the manuscript. All authors participated in the research, study design, manuscript preparation, and have approved the final manuscript.

## Funding

This study was supported by the Hunan Provincial Science and Technology Department Projects (2017SK50315, 2020SK2123), the Health and Family Planning Commission of Hunan Province Project (B20180484), the Science and Technology Bureau, Changsha Project (kq2004106), and the Hunan Brain Hospital Project (2017B03). These sources had no further role in determining the study design, data analyses, or preparation of the manuscript.

## Conflict of Interest

The authors declare that the research was conducted in the absence of any commercial or financial relationships that could be construed as a potential conflict of interest.

## Publisher's Note

All claims expressed in this article are solely those of the authors and do not necessarily represent those of their affiliated organizations, or those of the publisher, the editors and the reviewers. Any product that may be evaluated in this article, or claim that may be made by its manufacturer, is not guaranteed or endorsed by the publisher.
